# The Protocol of Choice for Treatment of Snake Bite

**DOI:** 10.1155/2016/7579069

**Published:** 2016-09-21

**Authors:** Afshin Mohammad Alizadeh, Hossein Hassanian-Moghaddam, Nasim Zamani, Mitra Rahimi, Mohammad Mashayekhian, Behrooz Hashemi Domeneh, Peyman Erfantalab, Ali Ostadi

**Affiliations:** ^1^Department of Bone Marrow Transplantation, Taleghani Hospital, Shahid Beheshti University of Medical Sciences, Tehran, Iran; ^2^Toxicological Research Center, Department of Clinical Toxicology, Loghman-Hakim Hospital, School of Medicine, Shahid Beheshti University of Medical Sciences, Tehran, Iran; ^3^Excellence Center of Clinical Toxicology, Iranian Ministry of Health, Tehran, Iran; ^4^Department of Emergency Medicine, School of Medicine, Iran University of Medical Sciences, Tehran, Iran; ^5^Department of Internal Medicine, School of Medicine, Tabriz University of Medical Sciences, Tabriz, Iran

## Abstract

The aim of the current study is to compare three different methods of treatment of snake bite to determine the most efficient one. To unify the protocol of snake bite treatment in our center, we retrospectively reviewed files of the snake-bitten patients who had been referred to us between 2010 and 2014. They were contacted for follow-up using phone calls. Demographic and on-arrival characteristics, protocol used for treatment (WHO/Haddad/GF), and outcome/complications were evaluated. Patients were entered into one of the protocol groups and compared. Of a total of 63 patients, 56 (89%) were males. Five, 19, and 28 patients were managed by Haddad, WHO, or GF protocols, respectively. Eleven patients had fallen into both GF and WHO protocols and were excluded. Serum sickness was significantly more common when WHO protocol was used while 100% of the compartment syndromes and 71% of deformities had been reported after GF protocol. The most important complications were considered to be deformity, compartment syndrome, and amputation and were more frequent after the use of WHO and GF protocols (23.1% versus 76.9%; none in Haddad; *P* = NS). Haddad protocol seems to be the best for treatment of snake-bitten patients in our region. However, this cannot be strictly concluded because of the limited sample size and nonsignificant *P* values.

## 1. Introduction

Snake bite is a common and very important health problem in many parts of the world including our country [1, 2]. Apart from the production of antivenom, snake envenomation shares all characteristics of a neglected tropical disease in Asia [3]. Snake bite has caused almost from 4.5 to 9.1 effect rate in each 100000 Iranian population and 67 deaths (0.1% mortality rate) during 2002 to 2011 [2]. Although mortality rate of snake bite is fairly low, the complications due to it or its treatment (including coagulopathies, renal and/or pulmonary failure, disseminated intravascular coagulopathy, hemorrhages, deformities, compartment syndrome, limb amputation, and serum sickness syndrome) are rather frequent [1, 4].

**Figure 1:**
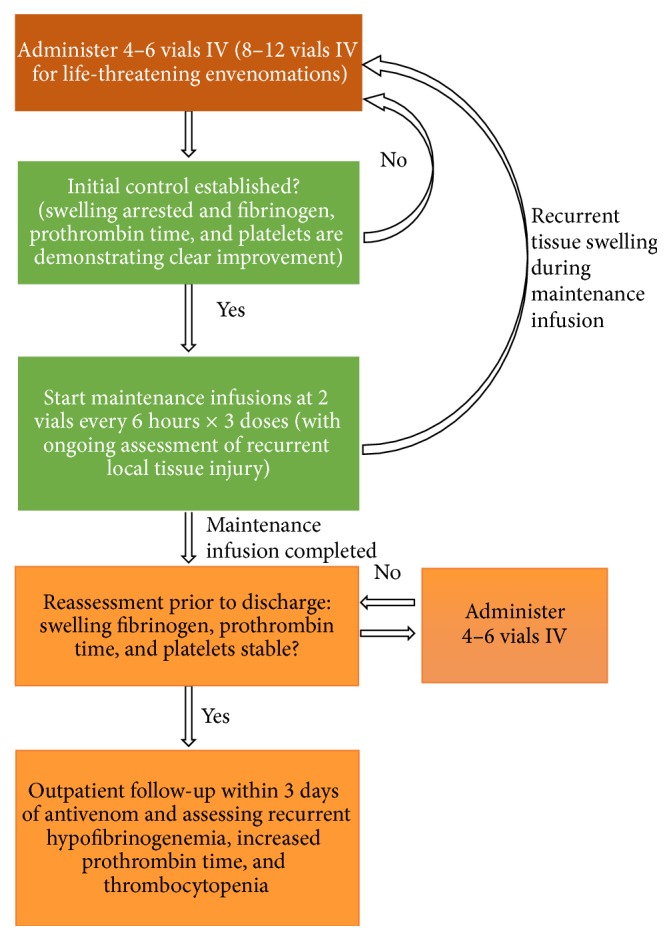
Flowchart of treatment of snake bite by* Goldfrank's Toxicologic Emergencies* textbook [6].

**Figure 2:**
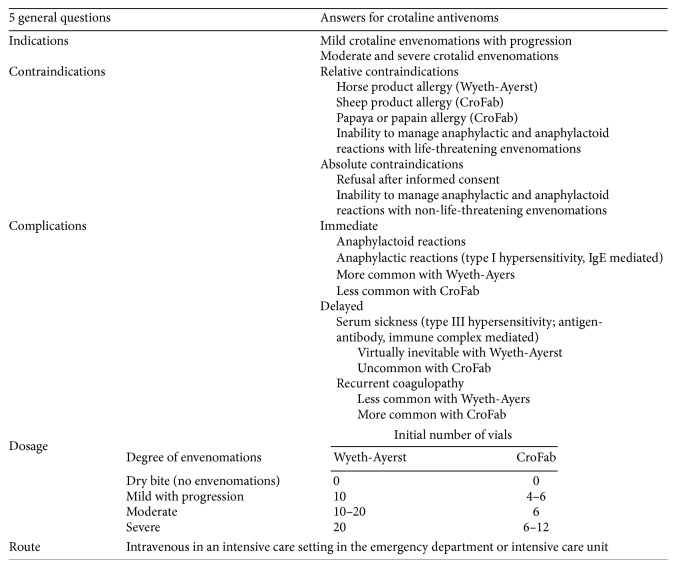
Flowchart of treatment of snake bite by* Haddad and Winchester's (Haddad) Clinical Management of Poisoning and Drug Overdose* textbook [7].

Different protocols exist to manage snake bite, some of the very commonly used ones of which are the protocols suggested by the World Health Organization (WHO),* Goldfrank's Toxicologic Emergencies* (GF) textbook (Figure 1), and* Haddad and Winchester's (Haddad) Clinical Management of Poisoning and Drug Overdose* textbook (Figure 2) [5–7].

Interestingly, these protocols are far different from each other regarding management of the patients and even in the determination of the severity of poisoning (Table 1) [6, 7]. They all have their own fans. No study has compared the efficacy of these protocols to determine the most efficient one with the least complications.

In Iran, of three types of antivenom, only polyvalent one is produced by the Razi Vaccine and Serum Research Institute. The polyvalent product can neutralize the venom of six different venomous snake species including* Naja naja oxiana*,* Pseudocerastes persicus fieldi*,* Echis carinatus*,* Vipera albicornuta*,* Vipera lebetina obtusa*, and* Agkistrodon halys* [2]. They are produced by plasma condensation and purification of immunized horses and contain 10 mLs of effective substance which can intravenously or intramuscularly be administered. Our center is a tertiary clinical toxicology center with an annual admission rate of about 30 to 40 snake-bitten patients. In a previous study from our center, two deaths were reported following venomous animals envenomation [8]. Five attending physicians of this center use different protocols of snake bite treatment (mostly GF and WHO) based on their personal favorite but not on the patients' clinical condition. In a try to unify the protocol of snake bite treatment in our center, we reviewed the files of the snake-bitten patients and compared the outcome and frequency of complications between them to determine which protocol was probably the best for the management of these patients.

## 2. Methods

Files of all patients who had been bitten by snakes and referred to a single tertiary toxicology center within five years (April 2010 to April 2014) were retrospectively evaluated. Data was extracted by a single abstractor. The data extracted included patients' demographics (age and sex), the site of snake bite, time elapsed between bite and hospital presentation, on-arrival signs and symptoms, treatment protocol used for the treatment of the patient (WHO versus Haddad versus GF), numbers of the vials given to each patient, complications during the hospital stay (development of cellulitis, compartment syndrome, fasciotomy, and limb amputation), complications developed after hospital discharge (fever, swelling, and redness for determination of cellulitis; fever, rash, and arthritis/arthralgia for serum sickness syndrome; and limb deformities), hospital stay, and final outcome of the patients (complete recovery, recovery with sequelae, or death). However, since WHO has no suggested specific protocol for our region, a modified WHO protocol focused on specific snakes of Iran (developed by Iranian Ministry of Health) is used in our country [5]. Compartment syndrome was confirmed by doppler ultrasonography in each case.

Two fellows reviewed all charts and determined if the patient had been managed by WHO, GF, or Haddad protocols. The criteria for assessing compliance to the treatment protocol were based on severity of envenomation defined in each protocol, number of used vials, and repetition of it during hospitalization course. In case they disagreed on one decision, a third expert (an attending physician) entered their decision making process and convinced them to reach the same decision. Finally, the experts agreed on all charts and their protocol. For their follow-up, the patients were contacted using phone calls.

Their main postdischarge complications were evaluated using a self-made questionnaire evaluating the development of serum sickness, cellulitis, and permanent complications such as deformity of the bitten limb. The patients were then entered into one of the protocol groups and compared regarding the treatment performed, complications developed, and final outcome.

The data was entered into statistical package for social sciences (SPSS) version 17 and analyzed using Student's* t*-test (mean difference) and Kruskal-Wallis* H* test (median difference) for continuous data and chi-square test (for categorical data). A *P* value less than 0.05 was considered to be statistically significant. The study was approved by the Local Ethics Committee of Shahid Beheshti University of Medical Sciences.

## 3. Results

A total of 147 viper-bitten patients had been referred to us during the study period. Of them, only 63 could be followed up by phone calls and 56 (89%) were males. Five, 19, and 28 patients were managed by Haddad, WHO, or GF protocols, respectively, while 11 had fallen into both GF and WHO protocols and were therefore excluded. In fact, due to the similarity of these two protocols in mild cases, we could not determine which protocol the treating physician had chosen and thus we excluded the patients. In the remaining 52 patients, 46 (88%) were males. The most common site of snake bite was fingers (24 patients; 46%) followed by feet (12 patients; 23%) and calves (5 patients; 10%). None of the patients were bitten in the head and neck. The most common signs/symptoms on presentation were swelling (51 patients; 98%) and pain (44 patients; 85%). The patients were considered to have mild, moderate, or severe envenomations according to the protocol applied for their treatment as this classification may significantly differ in different treatment protocols. Complications including serum sickness, deformity, compartment syndrome needing fasciotomy, amputation, necrosis, and neuropathy were detected in 10 (19.2%), 7 (13.5%), 4 (7.7%), 2 (3.8%), 2 (3.85), and 1 (1.9%) patients, respectively. Serum sickness was significantly (*P* = 0.04) more common when WHO protocol was applied (70% of all cases of serum sickness), while 100% of the compartment syndromes and 71% of all deformities had been reported after treatment with GF protocol. The most important complications were considered to be deformity, compartment syndrome, and amputation and were more frequent after use of WHO and GF protocols (23.1% versus 76.9%; none in Haddad; *P* = NS; Table 2).

## 4. Discussion

According to our results, although the sample size is limited, Haddad protocol seems to be the best method of snake bite treatment. It causes least important complications (deformity, compartment syndrome needing fasciotomy, and amputation) and even less serum sickness in comparison with the other two protocols. However, based on the number of the vials advised by each protocol, Haddad suggests the most invasive treatment. As shown in Table 2, the amount of recommended antivenom is significantly more in Haddad protocol.

Increasing amount of administrated antivenom usually increases the risk of serum sickness [9]. Haddad generally advises 10, 10–20, and more than 20 vials for mild, moderate, and severe envenomations, which is far beyond the vials recommended by GF (4–6 in each step before reconsideration) while having 3 to maximum 20 vials by WHO [5–7]. We think this is mainly due to the fact that the earlier the patients receive their antivenom, the faster they improve. Previous authors have also emphasized the protective role of early antivenom administration on the snake-bitten patients and its fair effects on their final outcome [10].

We believe that although administration of 4–6 vials and reconsideration of the patients according to the GF protocol (and somehow WHO protocol) prevent administration of excessive antivenom vials, it predisposes the patient to higher risk of insufficient vial administration in the early hours after bite which are the critical hours in patient management since the best results are withdrawn when the antivenom is initiated within 24 hours [11]. On the other hand, it seems that early administration of high numbers of vials—as suggested by Haddad—should predispose the patient to higher risk of later serum sickness syndrome; this was not supported by our study, a result that we could not explain.

## 5. Limitations of This Study

The retrospective nature of the study was definitely a limitation of the current study. Also, difference between the common snakes at the home of the textbooks and ours, difference in the antivenoms available in our country and theirs, and very few numbers of the studied patients who were even needed to be reduced to only 52 cases are possibly other limitations that should be considered in future studies. In fact most of our patients were shepherds and could not be followed up through phone calls. However, it should be mentioned that a possible strength of our study is that we used the same polyvalent antivenoms manufactured by a single factory for all patients and in all episodes.

Also, the occurrence of serum sickness might relate to the dose of antivenoms and their quality and it was unreasonable to find serum sickness more common in group of WHO protocol. This was however a finding of the current study that should be further investigated in the future studies. In conclusion, although Haddad's protocol seems to be the best for treatment of snake-bitten patients in our region, this cannot be strictly concluded because of the limited sample size. Further prospective studies on more sample sizes are warranted to determine the best protocol for snake-bitten patients in different regions.

## Figures and Tables

**Table 1:** Iranian-modified WHO diagram for management of snake bite.

Severity of envenomation	Signs/symptoms	Number of the vials that should be given
Mild	Local swelling without systemic signs/symptoms	3–5

Moderate	Extension of swelling with systemic signs/symptoms (paresthesia, nausea and vomiting, diarrhea, fatigue, lightheadedness, sweating, and chills) ± lab test abnormalities	6–10

Severe	Extension of swelling to all affected limb with systemic signs/symptoms (respiratory failure, shock, bleeding, loss of consciousness, fasciculation, and seizure) and severe lab test abnormalities	11–20

**Table 2:** Follow-up data on three common snakebite protocols (*n* = 52).

Variable	WHO *n* = 19	GF *n* = 28	Haddad *n* = 5	Sig.	Posttest
Antivenom used vials (min, max)	5 [2, 6] (2, 18)	5 [5, 8] (0, 30)	10 [10, 12] (10, 12)	.016	*P* = 0.013, Haddad-WHO^*∗*^ *P* = 0.021, GF-Haddad^*∗*^
Deformity *n* (%)	2 (10.5)	5 (17.9)	0	NS	—
Amputation *n* (%)	1 (5.3)	1 (3.6)	0	NS	—
Fasciotomy *n* (%)	0	4 (14.3)	0	NS	—
Necrosis *n* (%)	1 (5.3)	0	1 (20)	NS	—
Neuropathy *n* (%)	0	1 (3.6)	0	NS	—
Serum sickness *n* (%)	7 (36.8)	2 (7.1)	1 (20)	.04	*P* = 021, GF-WHO^*∗∗*^
Hospital stay (day) (min, max)	2 [1, 2] (1, 12)	3 [2, 4] (1, 9)	3 [1.5, 4.5] (1, 5)	.035	*P* = 0.035, WHO-GF^*∗*^

^*∗*^Using post hoc adjusted test. ^*∗∗*^Using Pearson chi-square.
